# Physiology and Therapeutic Potential of SK, H, and M Medium AfterHyperPolarization Ion Channels

**DOI:** 10.3389/fnmol.2021.658435

**Published:** 2021-06-03

**Authors:** Deepanjali Dwivedi, Upinder S. Bhalla

**Affiliations:** ^1^National Centre for Biological Sciences, Tata Institute of Fundamental Research, GKVK Campus, Bengaluru, India; ^2^Department of Neurobiology, Harvard Medical School, Boston, MA, United States; ^3^Stanley Center at the Broad, Cambridge, MA, United States

**Keywords:** ion channels, neuropsychaitric disorders, therapeutic targets, advances and challenges, SK, HCN and M channels

## Abstract

SK, HCN, and M channels are medium afterhyperpolarization (mAHP)-mediating ion channels. The three channels co-express in various brain regions, and their collective action strongly influences cellular excitability. However, significant diversity exists in the expression of channel isoforms in distinct brain regions and various subcellular compartments, which contributes to an equally diverse set of specific neuronal functions. The current review emphasizes the collective behavior of the three classes of mAHP channels and discusses how these channels function together although they play specialized roles. We discuss the biophysical properties of these channels, signaling pathways that influence the activity of the three mAHP channels, various chemical modulators that alter channel activity and their therapeutic potential in treating various neurological anomalies. Additionally, we discuss the role of mAHP channels in the pathophysiology of various neurological diseases and how their modulation can alleviate some of the symptoms.

## Introduction

The correct regulation of neuronal excitability is crucial for healthy brain functioning. The cooperative activity of depolarizing and hyperpolarizing ion channels shape a neuron’s firing activity. Potassium channels are the primary ion channels which mediate outward potassium currents to repolarize/hyperpolarize the membrane potential, thereby limiting neuronal excitability (Ranjan et al., 2019). Afterhyperopolarization mediated by diverse types of potassium channels repolarizes the membrane, limits spike width and amplitude, and controls firing activity, thus preventing neurotoxicity due to excessive firing (Zhang and McBain, 1995). Based on their activation kinetics, different potassium channels mediate afterhyperpolarization at distinct time scales and are categorized as fast afterhyperpolarization (activated in 1–5 s), medium afterhyperpolarization (mAHP, activated between 10–300 ms), and slow afterhyperpolarization (sAHP, activated between 0.5-multiple seconds) (Storm, 1990; Zhang and McBain, 1995). Multiple studies have indicated that hyperpolarization activated cyclic nucleotide (HCN) channels, voltage gated K^+^ channel 7 (K_v_7) and small conductance calcium activated potassium (SK) channels contribute to mAHP in neurons (Gu et al., 2005; Mateos-Aparicio et al., 2014). In the present review, we provide a detailed description of these mAHP-mediating ion channels. These channels control cellular excitability, and the anomalies associated with them can cause seizures, hyperactivity, and multiple neurological disorders. Thus, the pharmacological manipulation of these channels has therapeutic potential. The current review provides an insight into the biophysical properties of the channels, the kinetics of their various isoforms, their regional expression levels in the brain, and advancements in their therapeutic application for treating multiple neurological disorders.

## mAHP Channels and Their Isoforms

Medium afterhyperpolarization channels exhibit a diverse distribution throughout the nervous system. Herein, we discuss the individual properties of the three main categories of mAHP channels in detail.

Based on phylogenetic analysis, the SK channel family comprises SK1 (KCa2.1), SK2 (KCa2.2), and SK3 (KCa2.2) as well as a fourth channel, SK4 (KCa3.1, IK1), which performs a function that is comparable but unrelated to the functions of the other SK channels (Wei et al., 2005; Kuiper et al., 2012). SK channels are voltage insensitive and are activated solely by an increase of 0.5–1 μM in intracellular calcium (Ca^2+^) levels (Blatz and Magleby, 1986; Köhler et al., 1996; Sah, 1996; Hirschberg et al., 1999). An individual channel has a conductance of 10 pS and achieves its half activation at an intracellular calcium level of approximately 0.6 μM (Hirschberg et al., 1999). The time constant of channel activation is 5–15 ms, and the deactivation time is 30 ms (Xia et al., 1998; Oliver et al., 2000). Many channel isoforms are generated by alternative splicing. Among the three SK channels, SK1 undergoes maximum alternative splicing to yield at least 16 SK1 isoforms (Shmukler et al., 2001). The presence of multiple SK1 variants indicates the diversity of the roles of this channel. A study by Strassmaier et al., 2005 reported SK2-L (the longer isoform weighing 78 kDa) and SK2-S (the smaller isoform weighing 49KDa) as two novel isoforms. Additionally, Murthy et al., 2008 discovered a cytoplasmic variant of SK2, which lacks the transmembrane fractions S3, 4, 5, and 6 and mediates the downstream effects of cytokine activation. Another splice variant of the SK2 channel that carries three extra amino acids at the 3’ terminus, couples with α9/10 nicotinic acetylcholine receptors (nAchRs) at olivocochlear synapses in the cochlea and controls their activity (Scholl et al., 2014). Lastly, SK3-1B is a truncated isoform of SK3 that is known to represent 20–60% of the total SK3 present in the brain (Tomita et al., 2003; Shakkottai et al., 2004; Villalobos et al., 2004). These findings demonstrate the isoform-dependent heterogeneity of the SK channels. Different channel isoforms also exhibit distinct localization in various neuronal compartments or brain regions, thereby imparting specialized neuronal functions to different brain regions (Table 1).

**TABLE 1:** Summary of the regional expression of different mAHP channels in distinct brain regions and the specific subcellular and region-specific distribution of various channel isoforms.

**mAHP channel**	**Regions**	**References**	**Subcellular distribution**
SK	Hippocampus (CA1, CA2, CA3 pyramidal cells, DG neurons, Internueorns)	Zhang and McBain, 1995; Stocker and Pedarzani, 2000; Sailer et al., 2002; Allen et al., 2011	• SK1 and SK2 are the predominant channel isoforms present in the hippocampus and cortex, while SK3 is predominant in the striatum, medial habenular nucleus, locus coeruleus, dorsal raphe, and thalamus.
	Cortex (piriform cortex, entorhinal cortex, para-, pre- subiculur cortex	Schwindt et al., 1988; Criado-Marrero et al., 2014	Further, the two isoforms of SK2, SK2-S, and SK2-L also have a differential expression within a neuron. SK2-L is present in the postsynaptic density (PSD) while SK2-S localizes in extrasynaptic sites, when not in a multimeric complex with SK2-L (Allen et al., 2011).
	Cerebellum (Deep nuclei, purkinje cells, golgi cells, granule cells)	Stocker and Pedarzani, 2000; Walter et al., 2006	• Within the spinal cord, SK2 is present in all the α-motoneurons, while SK3 is preferentially present in small diameter α-motoneurons (Deardorff et al., 2013).
	Amygdala (Basolateral, Basomedial and Lateral amygdaloid nucleus)	Power and Sah, 2008	• Within the hippocampus, SK channels are present throughout a neuron, i.e., in soma, dendrites, and spines (Cai et al., 2004; Dwivedi et al., 2019). At spines, they are present in a complex with NMDARs, mGLuRs, and Calcium channels (Köhler et al., 1996; Bloodgood and Sabatini, 2007; Fakler and Adelman, 2008).
	Nucleus basilis neurons, Paraventricular neurons	Williams et al., 1997; Chen and Toney, 2009	• SK3 channels have presynaptic localization in the hippocampus, present in mossy fibers terminals but not in pyramidal layer (Obermair et al., 2003).
	Striatal cholinergic interneurons and Basal nuclei	Goldberg and Wilson, 2005; Bishop et al., 2010	• Within the brain, HCN1 is enriched in the neocortex, CA1 hippocampus, superior colliculus, fornix, superior colliculus, hypoglossal nucleus, cochlear nuclei, olfactory bulb, layer II and V in cortex and cerebellum.
	Olfactory system, Neocortex, septum, Brain stem	Habermann and Fischer, 1979; Stocker and Pedarzani, 2000	• Unlike HCN1, HCN2 has ubiquitous expression throughout the brain. High levels are observed in the cortex, hippocampus, globus pallidus, brain stem, retina, olfactory bulb, cerebellum, and thalamus.
	Striatum, Medial habenular nucleus, Locus coeruleus and dorsal raphe and Thalamus	Tacconi et al., 2001; Sailer et al., 2002	• Both HCN3 and HCN4 are relatively lesser in the brain. • HCN3 is present at moderately higher levels in the olfactory bulb, in the piriform cortex, preoptic area, hypothalamic regions, and cochlear nuclei.
HCN	Thalamus (Principal relay nuclei, Geniculate nuclei, Subthalamic nuclei)	Moosmang et al., 1999; Santoro et al., 2000; Notomi and Shigemoto, 2004; Abbas et al., 2006; Ying et al., 2011; Ding et al., 2016	• HCN4 is enriched especially in olfactory bulb, thalamus, fasciculus retroflexus, substantia nigra and habenula with low levels of expression in hippocampus and dentate granule cells (Moosmang et al., 1999; Santoro et al., 2000; Moosmang et al., 2001; Notomi and Shigemoto, 2004).
	Cortex (Neocortex, Piriform Cortex, Anterior Cingulate cortex)	Moosmang et al., 1999; Santoro et al., 2000; Lörincz et al., 2002; Notomi and Shigemoto, 2004; Gao et al., 2016	• Peripheral nervous system expresses all the HCN isoforms, but HCN1 levels are higher than other isoforms (Novella Romanelli et al., 2016).
	Hippocampus (CA1, CA3, Stratum Oriens, Stratum Radiatum, Granule cells)	Moosmang et al., 1999; Santoro et al., 2000; Lörincz et al., 2002; Notomi and Shigemoto, 2004	• In hippocampal and neocortex neurons, HCN channels had a proximal to distal dendritic gradient with limited somatic localization. They have a higher expression in the dendritic shafts than spines (Lörincz et al., 2002).
	Amygdala (Basolateral nucleus, central nucleus, lateral nucleus)	Moosmang et al., 1999; Santoro et al., 2000; Notomi and Shigemoto, 2004; Park et al., 2011	• In the medial perforant pathway, HCN1 channels are localized only on axons (Bender and Baram, 2008).
	Cerebellum (Molecular, Purkinje, Granule cell layer and deep cerebellar nuclei)	Moosmang et al., 1999; Santoro et al., 2000; Notomi and Shigemoto, 2004	• In medial superior olive neurons, HCN channels are present in axon initial segment (AIS) and control spike threshold (Ko et al., 2016).
	DRG, primary nociceptive neurons	Moosmang et al., 1999; Emery et al., 2011; Hou et al., 2015	• Within thalamus, HCN2 and HCN4 channels have opposite gradient such that HCN2 channels are more abundantly present in ventrobasal (VB) neurons while HCN4 channels are predominant in reticular thalamic neurons (RTN) neurons (Abbas et al., 2006).
	Hypothalamus (Preoptic nuclei, Ventromedial nuclei and mammillary nuclei)	Moosmang et al., 1999; Santoro et al., 2000; Notomi and Shigemoto, 2004	• K_v_7.2-K_v_7.5 are the primary isoforms which constitute the M currents in the brain.
	Brain Stem (Dorsal horn and Ventral horn)	Moosmang et al., 1999; Santoro et al., 2000; Notomi and Shigemoto, 2004	• M channels are primarily present in axon initial segment, nodes of Ranvier, and on unmyelinated axons (Devaux et al., 2004; Vervaeke et al., 2006).
	Habenula (Lateral and Medial habenula)	Moosmang et al., 1999; Santoro et al., 2000; Notomi and Shigemoto, 2004	• In CA1 hippocampal neurons, M channels are present in the perisomatic region regulating somatic excitability but not in distal dendrites (Hu et al., 2007).
K_v_7	Hippocampal (CA1, CA2, CA3 pyramidal cells, DG neurons, Internueorns)	Cooper et al., 2000; Saganich et al., 2001; Roche et al., 2002	• In the hippocampus, hilar polymorphic cells, and subiculum pyramidal cells, both K_v_7.2.and 7.3 were expressed. However, on mossy fiber bundles and neuropil of dentate hilus, CA3, CA1, and subiculum only K_v_7.2 channels were found (Cooper et al., 2000; Roche et al., 2002)
	Thalamus [Medial Geniculate nucleus, Ventral posterior thalamic complex, dorsal lateral geniculate (dLG)]	Saganich et al., 2001	• K_v_7.5 channels localize in synapses of auditory nuclei (Caminos et al., 2007).
	olfactory bulb (Granular, Mitral and Periglomerular cell layer)	Saganich et al., 2001	• In the sciatic nerve, the nodes of Ranvier of large fibers expressed only K_v_7.2. On the contrary, both K_v_7.2 and K_v_7.3 channel isoforms showed expression at nodes of small and intermediate-sized fibers (Schwarz et al., 2006).
	Habenula	Kang et al., 2017	• Mesencephalic dopaminergic neurons exclusively express K_v_7.4 channel isoform (Hansen et al., 2008).
	basal ganglia (Caudate/Putamen, accumbens nucleus, Globus Pallidus)	Saganich et al., 2001	
	amygdala,	Tober et al., 1996; Saganich et al., 2001	
	midbrain, hypothalamus, substantia nigra (Pars reticulate, Pars compacta), cerebellum (Granule, molecular, purkinje cell layer and deep nuclei)	Saganich et al., 2001	
	superior cervical ganglion cells, motor neurons, dorsal horn and spinal neurons	Saganich et al., 2001; Passmore et al., 2003; Rivera-Arconada and Lopez-Garcia, 2005	
	Visceral sensory neurons, DRG, nodose ganglia,	Passmore et al., 2003; Wladyka and Kunze, 2006; Linley et al., 2008	

Hyperpolarization-activated cation (I_h_) currents are mediated by HCN channels. These channels comprise four α subunits, encoded by four related genes, HCN 1, 2, 3, and 4 (Biel et al., 2009). All four isoforms of HCN channels have distinct activation kinetics. The HCN1 isoform exhibits the fastest activation, its V_1__/__2_ for activation lies between -90 and -70 mV, and tau for activation lies between 30 and 300 ms. The HCN2 isoform exhibits activation between 150 ms and 1 s while the HCN3 isoform exhibits activation between 250 and 400 ms. Both HCN2 and HCN4 are activated between -70 and -100 mV. HCN4 is the slowest to activate; its activation time is hundreds of milliseconds, and activation occurs at an extremely hyperpolarized potential of −140 mV (Biel et al., 2009). However, for human HCN channels, HCN2 and 3 have similar activation kinetics with HCN1 being fastest and HCN4 the slowest (Stieber et al., 2005). The four HCN isoforms exhibit a structural homology of approximately 60%. The major heterogeneity among the different isoforms originates from differences in the N and C termini of the channels (Santoro et al., 1998; Kaupp and Seifert, 2001). Different HCN isoforms assemble to form homotetramers or heterotetramers, thus conferring a range of neuronal functions that vary with the constituent subunits. *In vivo*, HCN1 and HCN2, but not HCN2 and HCN3, can form functional heteromers (Much et al., 2003). HCN channel activity is strongly regulated by changes in the levels of cyclic nucleotide monophosphates (cAMP/cGMP). HCN2 and HCN4 are strongly affected by cAMP levels, with moderate effect on HCN1 and none on HCN3 (Stieber et al., 2005). HCN isoforms can also assemble with different auxiliary subunits, such as KCNE2/minK-related peptide 1 (MiRP1), at the C terminus to form functional channels (Yu et al., 2001; Decher et al., 2003). In addition to KCNE, tetratricopeptide repeat-containing Rab8b–interacting protein (TRIP8b) is another regulatory subunit on HCN channels that binds to the CNBD and the N bundle loop on the C terminus. TRIP8b assists in surface targeting of the channel and regulation of activation kinetics (Porro et al., 2020). Phosphorylation of Ser^237^ on TRIP8B helps in its binding to the HCN channels (Foote et al., 2019). Furthermore, HCN channels undergo several post-translational modifications, which contribute to their functional heterogeneity and expression dynamics. For example, HCN channels (HCN1, HCN2, and HCN4 but not HCN3) undergo S-palmitoylation, which enhances their ability to form heteromeric channels with other isoforms and accessory proteins (Itoh et al., 2016). Under inflammation and chronic pain, HCN2 channels in dorsal root ganglion (DRG) neurons can undergo SUMOlytion, which increases their surface expression (Forster et al., 2020). N-linked glycosylation of HCN channels is also known to assist in the trafficking of channel proteins to the membrane (Much et al., 2003) (Table 1).

Voltage gated K^+^ channel 7 channels mediate voltage-activated potassium currents called M currents. These channels are encoded by the K_v_7.1–K_v_7.5 family of genes and exhibit ionic conductance in the range 1–8 pS, which varies among channel isoforms (Barrese et al., 2018). The channels exhibit slow activation (in the range 100–300 ms) and deactivation (in the range 100–450 ms) kinetics and remain open for prolonged periods (Chen and Johnston, 2004; Delmas and Brown, 2005; Hansen et al., 2008; Barrese et al., 2018). Like SK and HCN channels, different K_v_7 isoforms (except K_v_7.1) can also form homo or heteromers resulting in ion channels with different kinetics and regional expression, which mediate different regulatory functions (Jentsch, 2000). In the central and peripheral nervous system, K_v_7.2–7.5 isoforms are the primary mediators of M current, and K_v_7.2/7.3 heteromers constitute the predominant M current–mediating channels in neurons (Wang et al., 1998; Jentsch, 2000). Among all the K_v_7 isoforms, K_v_7.2 has the most splice variants with significantly different C termini but identical N termini (Nakamura et al., 1998). Two K_v_7.2 splice variants, namely Q2L and Q2S, have also been found. Q2S forms non-functional M channels in underdeveloped fetal brains, while Q2L forms functional M channels in developed neurons (Smith et al., 2001). Furthermore, homologous K_v_7.2 channel splice variants have been reported in the human brain. The variants named K2ΔL, K2KL, K2ΔKΔL, and K2ΔLMP. Among these K2KL and K2ΔL form functional channels in COS cells (Tinel et al., 1998).

## Structure of mAHP Channels

**FIGURE 1:**
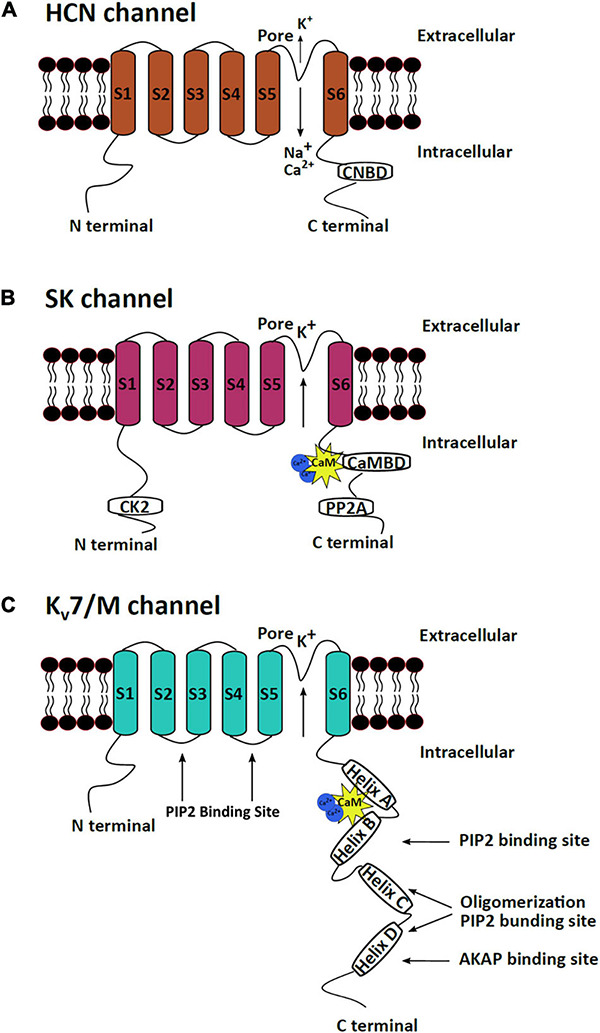
mAHP channel structures. The three mAHP channel subunits have a similar basic structure, which consist of six transmembrane segments, but significant heterogeneity is observed at the N and C termini. **(A)** HCN channels open in response to hyperpolarization and conduct a net inward current through the influx of Na^+^ and Ca^+^ and efflux of K^+^. The CNBD at the C terminus serves as the binding site for cAMP or cGMP, which regulates HCN channel activity. **(B)** SK channels are calcium-dependent potassium-conducting channels regulated by bound protein kinases and phosphatases at the N and C termini. **(C)** Kv7/M channels are voltage-sensitive potassium channels that are regulated by PIP2 and several protein kinases that bind to the C terminus of the protein.

The three mAHP channels have a high structural similarity with major structural differences in their N and C termini. All three channels are tetramers where each subunit has six transmembrane fragments. Different subunits assemble to form homo or heteromeric channels to create channels with varying kinetics and functions in distinct brain regions, thus imparting specialized neuronal activity (Ishii et al., 1997; Sailer et al., 2002; Monaghan et al., 2004; Strassmaier et al., 2005). Subunit (S) 4 of HCN and K_v_7 channels has positively charged lysine and arginine residues, which confers voltage sensitivity to the channels. S5 and S6 of the channels form the channel pore (Kaupp and Seifert, 2001). Both the N and C termini of the channels are cytoplasmic. The C termini of the three channels have binding sites for regulatory proteins. The C termini of SK and K_v_7 channels have calmodulin (CaM)-binding domain (CaMBD), while the HCN channels have a cyclic nucleotide-binding domain (CNBD) (Kaupp and Seifert, 2001; Barrese et al., 2018; Kshatri et al., 2018; Figure 1). CaM attached to SK and K_v_7 channels makes them sensitive to changes in intracellular calcium levels (Wainger et al., 2001). CNBD in HCN channels can bind to cAMP/cGMP, making HCN channels responsive to signaling pathways that modulate these secondary molecules. Binding of cAMP/cGMP to CNBD removes a conformational strain, which favors the activation of HCN channels (Wainger et al., 2001). CaMBD has positively charged and hydrophobic residues that facilitate CaM binding (Fanger et al., 1999; Zhang Y. et al., 2014). An intrinsically disordered region between S6 and CaMBD (R396–M412) on the SK channels assists in the binding of Ca^2+^ to CaM and causes channel activation (Zhang M. et al., 2013). CaM can achieve different conformations upon binding to different SK2 splice variants, thereby imparting varying calcium sensitivity to different channel isoforms (Zhang et al., 2012). The C termini of K_v_7 channels comprise four α-helices, which serve as a binding site for multiple regulatory molecules. The CaMBD of the K_v_7 channels is on helices A and B and serves as a binding site for both CaM and phosphatidylinositol 4,5-bisphosphate (PIP2) (Haitin and Attali, 2008; Barrese et al., 2018; Figure 1). Unlike most K^+^ channels (other than the inward rectifying potassium channels), HCN channels exhibit reverse polarity, making them capable of conducting cationic current in response to hyperpolarization instead of depolarization (Lee and MacKinnon, 2017). Cryo-electron microscopy has assisted in resolving the gating mechanism of HCN channels. A long S4 linker helix, in association with S4, S5, and S6, assists in stabilizing the closed state of the channel upon cellular depolarization (Lee and MacKinnon, 2017). The S4_C–term_ and S5_N–term_ mediate the interaction between the voltage-sensitive and pore domains of the channel, which keeps the channel closed during depolarization. However, upon hyperpolarization, the long S4 helix undergoes displacement, which opens the HCN channel (Lee and MacKinnon, 2017; Flynn and Zagotta, 2018).

The C termini of all the M channel isoforms exhibit a conserved A domain, which along with a proximal part of the B domain at the C terminal, assists in subunit assembly (Schwake et al., 2000, 2003; Robbins, 2001; Maljevic et al., 2003). The N termini of all M channel isoforms exhibit a high level of homology, while the C termini vary in lengths (Haitin and Attali, 2008). One CaM molecule per subunit is constitutively bound to the proximal C terminus of K_v_7 channels and is crucial for proper channel folding (Wiener et al., 2008). The binding of CaM on helix A and B also assists in the heteromeric assembly of K_v_7.2 and 3 (Liu and Devaux, 2014). In addition to the CaMBD, the C termini of K_v_7 channels also contain sites for binding other modulatory proteins such as kinases and scaffolding proteins (Delmas and Brown, 2005). Phosphorylation of specific serine residues in the PIP2 binding sites on Kv7 channels alters the PIP2-binding efficiencies of the sites and modulates channel activity (Salzer et al., 2017; Figure 1).

Expression and kinetics of mAHP channels are additionally regulated by some auxiliary/accessory proteins. Both K_v_7 and HCN channels interact with specific auxiliary proteins of the KCNE family, which alter their activation kinetics (Decher et al., 2003; Roura-Ferrer et al., 2009). KCNEs are single-subunit transmembrane proteins that can modulate both HCN and K_v_7 channels. KCNE 1, 3, and 4 interact with K_v_7 channels; however, only KCNE2 enhances the activity of HCN1, HCN2, and HCN4 (Decher et al., 2003). Deletion of KCNE2 reduces neuronal HCN1 and HCN2 levels, indicating that it also assists in surface targeting (Ying et al., 2012). Interaction with KCNE1 leads to slower inactivation and increased M current amplitudes (Jentsch, 2000). Furthermore, a single KCNE protein can have differential effects on different K_v_7 isoforms. For example, when KCNE3 interacts with K_v_7.2 channels, it can cause constitutive activation. By contrast, when KCNE3 binds to K_v_7.4 channels, it causes inhibition (Jentsch, 2000). Multiple KCNE proteins can complex with K_v_7 channels and exhibit intricate regulation of the activity of the channels (Lundby et al., 2010; Wrobel et al., 2012).

Therefore, as explained, the three mAHP channels have a similar basic structure. However, because of differences in their N and C termini, they can form heteromers with different channel isoforms and can be regulated by multiple regulatory elements. Together, these differences contribute significantly to heterogeneity in the kinetics and functions of the three mAHP channels.

## Endogenous Regulators of the mAHP Channel Activity

### Calcium

Calcium directly or indirectly regulates all three mAHP channels. CaM bound to the SK and K_v_7 channels sensitizes them to intracellular calcium levels (Fanger et al., 1999; Schumacher et al., 2001; Adelman, 2016). Intracellular calcium levels increase because of calcium influx through voltage-dependent calcium channels, through ionotropic glutamate receptors, such as NMDARs and AMPARs, and calcium-induced calcium release (CICR) from intracellular calcium reserves (Brennan et al., 2008). In CA1 hippocampal pyramidal neurons, L-type calcium channels in the soma and R-type calcium channels in the dendrites are structurally coupled to SK channels (Bloodgood and Sabatini, 2007). By contrast, P/Q-type calcium channels in Purkinje cells are functionally, but not structurally, coupled to SK channels. Calcium entry through P/Q-type Ca^2+^ channels leads to CICR, which activates SK channels (Marrion and Tavalin, 1998; Bloodgood and Sabatini, 2007). Furthermore, in outer hair cells, nAChRs are also coupled to SK channels. Ca^2+^ influx through nAChRs causes SK channel activation, which hyperpolarizes the outer hair cells (Oliver et al., 2000, 2001). Additionally, in the dendritic spines of hippocampal neurons, metabotropic glutamate receptor (mGluR5) and NMDARs are coupled to SK channels (Lin et al., 2008; Ngo-Anh et al., 2008; García-Negredo et al., 2014). Such functional coupling between ionotropic and metabotropic channels to SK channels has led researchers to propose the presence of microdomains on the cellular membrane. In these microdomains, these channels, along with different membranal and cytoplasmic effector molecules, are in proximity with each other and jointly regulate neuronal functioning (Blackstone and Sheng, 2002; Fakler and Adelman, 2008). Increased intracellular calcium via CaM can increase the SK channel activity with a tau of ∼5–10 ms. Such fast Ca^2+^ regulation is possible as CaM is constitutively bound to the channel (Adelman, 2016). Using cryo electron microscopy Lee and MacKinnon (2018) have shown that four CaM molecules can bind to one SK channel tetramer (one CaM/subunit). The C lobe of CaM is attached to the channel in the absence of Ca^2+^. However, Ca^2+^ binding to the N lobe of CaM produces a conformational change, which results in rearrangement of the S6 of SK channel further opening the channel pore (Lee and MacKinnon, 2018).

**FIGURE 2:**
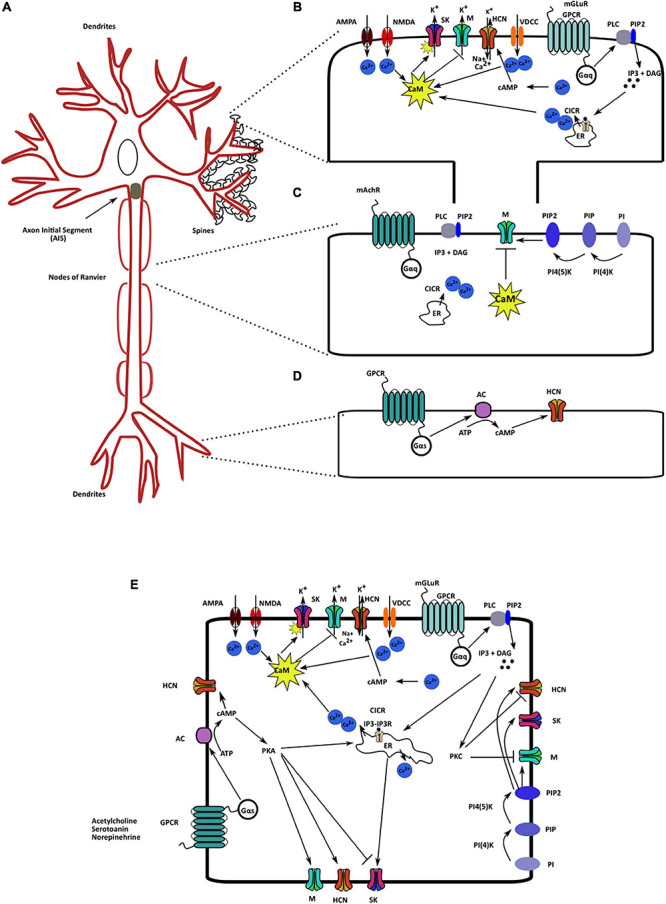
Localization and regulatory pathways of the mAHP channels. **(A)** A neuron schematic illustrating the localization of the three mAHP channels. SK channels are localized on the spines and modulate synaptic response. K_v_7/M channels present in the axon initial segment and node of Ranvier control cellular resting membrane potential (RMP) and input resistance (IR). HCN channels in hippocampal neurons are present as an increasing gradient from proximal to distal dendrites. **(B)** SK channels present on the spines are regulated by calcium influx from multiple sources; the primary sources include voltage-dependent calcium channels, ionotropic and metabotropic glutamate receptors, and CICR. **(C).** PIP2 levels and intracellular calcium via CaM serve as the most critical K_v_7/M channel regulators. **(D)** Changes in intracellular cAMP levels augment HCN channel activity through cAMP binding on the CNBD on the channel. **(E)** Various signaling and regulatory proteins co-exist in the intracellular milieu, modulating mAHP channels and regulating cellular excitability.

Increased intracellular calcium levels can also upregulate adenylyl cyclase activity, which increases intracellular cAMP and augments HCN channel activity (Halls and Cooper, 2011; Neymotin et al., 2016). Contrary to its effect on SK and HCN channels, increased intracellular calcium inhibits K_v_7 channels (Tobelaim et al., 2017). Calcium-free CaM is bound to both helices A and B at the C terminus of the K_v_7 channels. In this configuration, it assists in PIP2 binding, which is crucial for K_v_7 activation. An increase in intracellular calcium levels causes a structural rearrangement such that the calcium-bound CaM remains bound to helix B only. This rearrangement reduces PIP2 binding affinity, which reduces the opening probability of K_v_7.2–K_v_7.5 but not of K_v_7.1 channels (Barrese et al., 2018). In the case of K_v_7.1 channel, PIP2 and calcium-bound CaM share an overlying binding site. Thus, upon PIP2 depletion, calcium-bound CaM can bind to Kv7.1, mimic the PIP2 binding effect, and assist in channel activation (Tobelaim et al., 2017). Neurotransmitters, such as bradykinin, are known to regulate M channels via the CaM pathway (Gamper and Shapiro, 2003; Gomis-Perez et al., 2017) (Figure 2). Additionally, the βγ subunits of G proteins are also known to regulate K_v_7.4 channel activity by increasing its opening probability. They stabilize the coupling between PIP2 and K_v_7.4, thereby mediating their increased activation (Povstyan et al., 2017). Thus, calcium, through CaM directly activates SK channels and inhibits K_v_7 channels. Increased intracellular calcium levels increase HCN channel function by increasing cAMP levels.

### cAMP/cGMP

Similar to calcium, cyclic nucleotide monophosphates are also critical regulatory molecules for mAHP channels. Binding of cAMP/cGMP to the CNBD on HCN channels is one of the primary mechanisms underlying the regulation of HCN channel activity. The binding of cAMP to HCN channels is activity-dependent; hence, an increase in channel activity increases cAMP affinity to the channel (Wang et al., 2002). The binding of cAMP to the CNBD on HCN channels stabilizes the open channel conformation, which provides prolonged activation of HCN channels and facilitates neuronal excitability and rhythmic activity (Wang et al., 2002). Binding of cAMP to HCN channel can produce a 20 mV shift in their activation curve. However, cAMP binding exhibits variable effects on different HCN channel isoforms. cAMP binding does not affect HCN3, moderately affects HCN1 (shifts the activation curve by 2–4 mV), and strongly activates HCN2 and HCN4 channels (Stieber et al., 2005; Biel et al., 2009). Notably, because of the differential distribution of channel isoforms in various brain regions, changes in cAMP levels exhibit differential effects on the neuronal activity of distinct brain regions. Additionally, other cyclic nucleotides such as cUMP and cCMP (activates HCN2 and HCN4, but not HNC1 and HCN3) can also activate HCN channels (Zong et al., 2012; He et al., 2014).

G protein-coupled receptor activity causes cAMP production through the catalytic action of adenylyl cyclase. cGMP is produced downstream of the nitric oxide signaling pathway. Nitric oxide binds to and increases the catalytic activity of soluble guanylyl cyclase (Wilson and Garthwaite, 2010). Both cAMP and cGMP have the same binding site on HCN channels although they are produced through different signaling pathways. However, binding affinity of cAMP to HCN channels is ∼10–100 folds greater than that of cGMP (Biel et al., 2009). Neuromodulators such as acetylcholine, noradrenaline, serotonin, and epinephrine increase HCN channel activity by increasing intracellular cAMP levels (McCormick and Pape, 1990; Wilson and Garthwaite, 2010). However, changes in intracellular cAMP levels also increase K_v_7 channel activity (Schroeder et al., 1998).

Cyclic nucleotide monophosphates levels positively affect K_v_7 channel opening. Increased cAMP levels cause PKA-dependent phosphorylation of K_v_7.2 channels at the N terminus, which increases the channel conductance (Schroeder et al., 1998). Increasing cAMP levels also increase calcium influx, which in turn affects all the three mAHP channels (Konieczny et al., 2017; Figure 2). Thus, increased intracellular cAMP levels can produce larger mAHPs through their positive effects on the three mAHP channels. The aforementioned effect can function as a feedback mechanism to control neuronal excitability.

### PIP2

Like cAMP and cGMP, an increase in PIP2 levels also activates the three mAHP channels. PIP2 binding stabilizes the channels’ open configuration, which leads to increased conductance (Pian et al., 2006; Brown et al., 2007; Zhang Y. et al., 2014). This interaction between PIP2 and HCN/K_v_7 channels results from an electrostatic interaction between PIP2 head groups and the channel (Oliver et al., 2004; Zolles et al., 2006). Biological processes, such as the activation of muscarinic receptors that results in Gq signaling and increased PLC activity, consume PIP2 to produced IP_3_ and DAG. This causes PIP2 depletion, which results in a reduction in M and SK currents (Brown et al., 2007; Zhang M. et al., 2014). However, as a feedback mechanism, increased production of IP_3_ also increases calcium influx by CICR. Neuronal Ca^2+^ sensor I can sense an increase in calcium influx, which then stimulates the activity of PI4K and replenishes depleted PIP2 levels (Hernandez et al., 2008; Carver and Shapiro, 2018). Increased metabotropic Ach receptor (mAChR) stimulation in the dentate gyrus cells increases PIP2 synthesis as well as M currents. By contrast, in CA1 cells, the same mechanism causes PIP2 depletion and reduces M currents. Such opposing actions highlight the complexity and pleiotropic effects of the intracellular signaling cascade on M channels (Carver and Shapiro, 2018). Both mAChr and bradykinin cause a reduction in M and SK currents by the direct depletion of PIP2 levels. PIP2 binding affinity for K_v_7 is different for different K_v_7 isoforms. The affinity is highest for K_v_7.3, followed by that of K_v_7.2 and K_v_7.4 (Li et al., 2005). Different heteromers of different K_v_7 isoforms have affinities that are intermediate to those of their constitutive channels (Li et al., 2005). The PIP2 binding site on SK channels is in the vicinity of the CaMBD, and CK2-dependent phosphorylation of the CaMBD also reduces PIP2 binding affinity and SK channel activity (Zhang Y. et al., 2014). However, the PIP2 binding domain on HCN channels is not in proximity with the CNBD; consequently, both PIP2 and cAMP can separately regulate HCN activity (Zolles et al., 2006). PIP2 can produce a 20mV positive shift in the activation curve of HCN channels (He et al., 2014; Figure 2). Therefore, PIP2, in addition to being crucial for K_v_7 channel activation, is a positive regulator for the other mAHP channels.

### Protein Kinases

Protein kinases and phosphatases coupled to SK and K_v_7 can serve as additional channel activity regulators. CK2 bound to the C termini of SK channels phosphorylates CaM and reduces its calcium sensitivity, which favors reduced SK channel activity. By contrast, CK2 bound to the N terminus of K_v_7 phosphorylates CaM and strengthens its binding to the channel, causing increased channel activity (Bildl et al., 2004). CK2 kinase functioning is checked by coupled phosphatases, namely PP2A for SK and PP1 for K_v_7 channels (Maingret et al., 2008; Adelman et al., 2012; Kang et al., 2014). Kinase- and phosphatase-dependent regulation of SK channels modulate mGluR long-term potentiation (LTP) in hippocampal CA1 (Sourdet et al., 2003). Identical phosphoregulation of SK channels modulates LTP between parallel fibers and Purkinje cells in the cerebellum (Belmeguenai et al., 2010). Additionally, CK2 is enriched in the post synaptic density (PSD), where it can regulate NMDAR and AMPAR, which are also functionally coupled to SK channels (Castello et al., 2017). Thus, CK2 can directly (as bound to SK channel) and indirectly (via NMDAR and AMPAR) modulate SK channels, thereby regulating synaptic receptor functions.

In addition to CK2, PKA is another critical protein kinase that can regulate all the three mAHP channels. cAMP-PKA signaling can regulate the surface expression of SK channels. A high level of PKA activity reduces SK channels surface expression, while reduced PKA activity increases expression levels (Abiraman et al., 2016). A PKA-dependent decrease in SK channels surface expression levels facilitates NMDAR-mediated LTP induction in CA1 hippocampal neurons (Lin et al., 2008; Abiraman et al., 2016). In smooth muscle cells, increased cAMP-PKA activity facilitates K_v_7.5 and K_v_7.4 isoform activity (Mani et al., 2016). Similarly, in DRG neurons and hippocampal mossy fibers, PKA augments HCN channel activity (Mellor et al., 2002; Cheng and Zhou, 2013; Figure 2). PKC dependent phosphorylation of M channels increases their activity. However, under mGluR stimulation, A-kinase anchoring protein (AKAP) binds to PKC and reduces the accessibility of the PKC kinase site, resulting in a decrease in M current (Delmas and Brown, 2005; Kreir et al., 2019). In hippocampal and anterior cingulate cortex neurons, an increase in PLC–PKC activity via the mGluR signaling pathway also reduces HCN1 channel expression and HCN currents (Williams et al., 2015; Gao et al., 2016). Additionally, other signaling pathways that affect the above mentioned protein kinases can modulate the mAHP channels and regulate synaptic and cellular functions.

We have described the key regulatory molecules for the mAHP channels. Some of these regulators have a more significant impact on the activity of one type mAHP channel than on the activities of the other types; however, considerable overlap and common signaling pathways can affect all three mAHP channels. The isoform-specific effects of these regulators and the differential distribution of the mAHP channels can provide specialized neuronal functions in distinct brain regions.

## Functions of mAHP Channels in Regulating Intrinsic Cellular and Network Properties

### Intrinsic Cellular Properties

The diverse functionality of mAHP channels make them critical factors in various neurological diseases. mAHP channels play a crucial role in controlling neuronal excitability. Increased mAHP channel activity reduces the firing threshold for a cell, stabilizes RMP, and limits firing activity, thus controlling intrinsic neuronal excitability. Increased intracellular calcium (SK channels) and voltage change (HCN and M channels) cause channel activation. M and HCN channels, but not SK channels, are active at the RMP of the cell; hence, they strongly influence the RMP and IR of the cell. HCN channels specifically play a key role in stabilizing cellular RMP (Lupica et al., 2001). However, because of differences in the localization of the channels, M channels affect axonal RMP most significantly, while HCN channels control somatic and dendritic RMPs (Hu and Bean, 2018). Activation of M and SK channels also leads to spike frequency adaptation, which reduces net spiking output from a neuron (Ha and Cheong, 2017). Spike frequency adaptation is a progressive decline in the interspike interval in a spike train produced under sustained depolarization. Thus, the combined effects of the three mAHP channels control various intrinsic cellular properties and neuronal responses to input stimuli.

### Spike Generation in Soma and Dendrites

Specialized localization of M, HCN, and SK channels in different neuronal compartments regulate local neuronal properties. In general, activation of mAHP channels reduces excitability. However, in Layer 5 cortical cells, SK channels have a differential effect on excitability in the soma compared with that on apical dendrites (Bock and Stuart, 2016). SK channel activation in the soma reduces action potential output, while SK channel activation in dendrites increases dendritic spike generation. This differential effect is a result of the functional coupling of SK channels in apical dendrites to R-type calcium channels. Consequently, SK channel activation increases Ca^2+^ influx through R-type calcium channels, reduces the NMDAR-mediated spike threshold, and increases dendritic excitability (Bock and Stuart, 2016; Bock et al., 2019). M channels specifically regulate axonal properties because M channels are localized in axon initial segment (AIS), which enables them to regulate axonal excitability, axonal plasticity, and interneuronal signal transmission (Lezmy et al., 2017). In hippocampal neurons, HCN channels exhibit a proximal to distal dendritic gradient. Consequently, they strongly influence proximal to distal dendritic computation and hippocampal-specific learning and memory tasks (Berger et al., 2003). HCN1 channel activity is also crucial for Purkinje cell dendritic integration and assists in motor-dependent memory formation and coordination (Nolan et al., 2003).

### Synaptic and Network Activity Patterning

In addition to controlling intrinsic cellular properties, mAHP channels regulate neuronal synaptic activity, oscillatory activity of various neurons and network rhythms (Llinas and Jahnsen, 1982; Ludwig et al., 2003). Dendritic M and HCN activation produce a shunt inhibition on incoming excitatory post synaptic currents (EPSCs), thus reducing their amplitude and duration. This causes an increased threshold to spike and reduces EPSC integration (Berger et al., 2003). Presynaptic M and HCN channel activation control the paired-pulse ratio in the calyx of Held and sIPSC frequency in the amygdala, respectively (Martire et al., 2004; Huang and Trussell, 2011; Park et al., 2011). In Layer 3 EC neurons, presynaptic HCN channels reduce exocytosis of glutamate; hence, blocking presynaptic HCN channels produces an increase in miniature excitatory postsynaptic current frequency (Huang et al., 2018). SK channels in nucleus reticularis thalamic neurons and locus coeruleus (LC) control their pacemaker activity, thus driving network oscillations (Matschke et al., 2018). Rhythmic burst firing in nucleus reticularis thalamic neurons dependent on SK channels were reported to generate spindle waves (7–12 Hz) (Bal and Mccormick, 1993). SK channels, with T-type calcium channels in nucleus reticularis thalamic neurons, are responsible for sleep-related oscillations. SK2 channel knock outs exhibit a reduction in low-frequency rhythms in non–rapid-eye-movement sleep and disrupted sleep (Cueni et al., 2008). HCN channels control the circadian rhythm in suprachiasmatic neurons (Akasu et al., 1993; Atkinson et al., 2011), modulate hippocampal theta rhythms by controlling the firing activity of septohippocampal GABAergic neurons and stellate neurons of the entorhinal cortex (Dickson et al., 2000; Fransén et al., 2004; Xu et al., 2004); furthermore, HCN channels control the pace-making activity of globus pallidus neurons (Chen et al., 2015). Neurotransmitters such as noradrenaline and serotonin can further modulate these oscillatory activities through their effect on HCN channels (McCormick and Pape, 1990; Maingret et al., 2008; Giessel and Sabatini, 2010). Thus, mAHP channels play a significant role in shaping network rhythms, which affects the cognitive state and functioning of animals.

## Therapeutic Potentials of mAHP Modulators

As discussed, mAHP channels are crucial in regulating essential neuronal functioning. Consequently, mutations that directly or indirectly affect these ion channels lead to severe neurological defects. Herein, we provide an in-depth account of various clinical disorders where altered functioning of mAHP channels contributes to multiple symptoms.

### Ischemia

Ischemia is a commonly occurring form of brain damage, where the brain experiences a partial reduction or complete stoppage in blood supply because of cardiac arrest or stroke. Alterations in mAHP channel activity can either aggravate or circumvent ischemic damage. During an ischemic episode, the brain undergoes excitotoxic damage because of elevated extracellular glutamate levels that lead to increased calcium influx through NMDARs (Lo et al., 2003). Additionally, studies have shown that an ischemic attack causes a reduction in SK and an increase in HCN channel activity. This change further increases neuronal activity, thereby aggravating neuronal damage. A specific example of this is CA1 hippocampal neurons, where after an ischemic episode, SK2 channels decouple from NMDARs in the PSD, aggravating increased extracellular glutamate-induced damage to neurons (Allen et al., 2011). These neurons also undergo an increase in HCN1 and HCN2 activity levels after a transient ischemic insult. The increase in HCN channel activity decays after 4 days of the ischemic incidence, which contributes to the initial excitotoxic damage associated with ischemia. Both SK channel activators and HCN channel antagonists have shown neuroprotective action against ischemic attacks (Table 2). In CA3 cells, as a natural defense mechanism, transient ischemic insult activates SK currents, which provide a neuroprotective effect (Tanabe et al., 1999). In HT22 cells, glutamate-mediated oxidative stress increases mitochondrial SK2 channel activity, which exhibits a neuroprotective effect on these cells (Honrath et al., 2017; Krabbendam et al., 2018). Increasing SK channel activity by using 1-EBIO (SK channel agonist) increases the effect of Mg^2+^ blocking on NMDARs and prevents glutamate-mediated excitotoxic damage (Allen et al., 2011). The use of the HCN blocker ZD7288 and the M channel activator retigabine soon after ischemic damage (within 0–6 h) prevents excessive activation of postsynaptic NMDARs, prevents LTP deficits, and provides neuroprotection against oxygen or glucose deprivation in organotypic hippocampal cultures and *in vivo* ischemic models (Boscia et al., 2006; He et al., 2014; Bierbower et al., 2015; Diao et al., 2019; Chen et al., 2020; Table 2). Astrocytes also respond to ischemia to prevent neurotoxicity. HCN1 and HCN2 levels increase in astrocytes after 4 days and up to 2 weeks, contributing to long-term compensatory or neuroprotective effects (Honsa et al., 2014; Park et al., 2019). Thus, SK and M activators and HCN channel inhibitors are promising therapeutic targets for neuroprotection against ischemic insult (Table 3).

**TABLE 2:** Summary of different mAHP channels’ inhibitors and activators.

**mAHP channel**	**Modulator**	**Effect**	**References**
**SK**	**Inhibitors** – bee venom apamin (extracted from *Apis mellifera)* – scorpion venom toxin scyllatoxin (extracted from scorpion *Leiurus quinquestriatus hebraeus*) – d-tubocurarine (extracted from plant *Chondrodendron tomentosum)* – Chemical inhibitors like calcium chelators (EGTA, EDTA, and BAPTA) and cadmium – Artificial blocker, NS8593	• Both apamin and scyllatoxin block the pore region between S5 and S6, thereby inhibiting current flow through SK channels. Low levels of apamin can block SK2 channels (IC_50_ 62 pM), while apamin levels as high as 100 nm cannot block SK1 channels • They bind through their basic amino acid, arginine, on the negatively charged residues in the pore region of SK channels. • d-tubocurarine can block SK2 channels at a considerably lower level (IC_50_ 5.4 μM) than SK1 (IC_50_ 354.3 μM) channels. SK3 has intermediate sensitivity to apamin and d-tubocurarine. • Structural heterogeneity and minor differences in the amino acids in the pore region of different SK isoforms leads to variation in sensitivity to apamin and d-tubocurarine binding. • NS8593 is a highly potent negative modulator of SK channels. It acts by increasing the response threshold of SK channels to calcium. The drug can also cross the blood–brain barrier, which increases its clinical use to target SK channels in the brain.	Park, 1994; Ishii et al., 1997; Fanger et al., 2001; Köhler et al., 1996; Nolting et al., 2007; Sørensen et al., 2008
	**Activators** – Chlorzoxazone – 1-EBIO – NS 309 – SKS-11 and SKS-14 – CyPPA – Riluzole.	• 1-EBIO (EC_50_ of 630 μM for SK1, 500 μM to 1 mM for SK2, and 170 μM to 1 mM for SK3) increases calcium sensitivity, which increases SK channel activity. • 1-EBIO activates SK channels by binding both to the CaMBD and to CaM on the channel’s C terminus. • NS309 (EC_50_ 10–20 nM for KCa3.1 and approximately 600 nM for KCa2 channels) shares the same binding site as 1-EBIO, riluzole and CyPPA. • SKS-11 and SKS-14 are highly potent SK activators. These chemicals share the same binding site as 1-EBIO and NS309 and bind through strong electrostatic bonds with the channel and lock them in an open state. • CyPPA has a high specificity toward SK2 and SK3 channels but not SK1 channels. Isoform-specific SK channel modulators facilitate targeting specific regions depending on the isoform expression.	Brown et al., 2017; Zhang et al., 2012; Schumacher et al., 2001; Christophersen and Wulff, 2015; Cho et al., 2018; Hougaard et al., 2007; Nam et al., 2017
**HCN**	**Inhibitors** – Cesium and ZD7288. – α2-adrenoceptor agonists, such as clonidine, dexmedetomidine, and guanabenz – Ivabradine, Clonidine – Loperamide – Capsazepine	• Cesium and ZD7288 (IC_50_ approximately 10 μM) are specific blockers of HCN channels. The IC_50_ values for blocking the four channel isoforms are similar owing to a high structural homogeneity between the different isoforms. • These blockers produce a hyperpolarizing shift in the activation voltage of HCN channels and reduce maximal channel conductance. • Alanine 425 and isoleucine 432 on S6 are crucial for binding of ZD7288 to HCN channels. • Ivabradine (IC_50_ approximately 1–2 μM) and clonidine (IC_50_ approximately 10 μM) are specific HCN4 and HCN1 blockers, which bind to the intracellular side of the channel. • Loperamide (IC_50_ approximately 4.9–11 μM) acts extracellularly and binds to the S1-S2 region on HCN1 channels. • Capsazepine (IC_50_ approximately 8 μM) is another specific HCN1 and HCN2 channel blocker that blocks HCN channels in a dose-dependent manner	Cheng et al., 2007; Tanguay et al., 2019; Won et al., 2019; Bucchi et al., 2002; Vasilyev et al., 2007; Gill et al., 2004; Zhao et al., 2019
	**Activators** – Tanshinone IIA, extracted from *Salvia miltiorrhiza* – Fisetin – Lamotrigine and Gabapentin	• Tanshinone has a higher specificity for HCN2 than for the other isoforms. • Fisetin (EC_50_ approximately 2 μM) is a flavonoid activator of HCN2 channels, which shifts the channel V_1/2_ toward depolarized potentials. It binds to the CNBD on the channel and shares the same binding pocket as cAMP. • Lamotrigine and Gabapentin increase HCN current amplitude but do not affect channel activation or deactivation kinetics • Gabapentin is used to treat epilepsy and specifically affects the HCN4 isoform with small effect on other HCN isoforms.	Shang et al., 2012; Carlson et al., 2013; Surges et al., 2003; Peng et al., 2010; Tae et al., 2017
**M**	**Inhibitors** – XE991 and linopirdine – Drugs such as chromanol 293B, HMR1556, L-768, 673, JNJ282, and JNJ303	• XE991 (IC_50_ 0.8 μM) and linopirdine (IC_50_ 5 μM) are non–isoform-specific K_v_7 blockers. • Chromanol 293B, HMR1556, L-768, 673, JNJ282, and JNJ303 can block only K_v_7.1 when bound to KCNE1 and KCNE3.	Barrese et al., 2018
	**Activators** – Retigabine – Gabapentin – zinc pyrithione – Flupirtine – SCR2682,	• Retigabine is a K_v_7 channel activator that produces a hyperpolarization shift in the activation voltage, accelerates the activation kinetics of the channels, and slows the channel deactivation rate, • Retigabine can activate K_v_7.2, 7.3, 7.4, and 7.5 but not K_v_7.1. • Retigabine binds at the pore regions, where an interaction with a tryptophan residue at the cytoplasmic site of the S5 domain is crucial, thereby stabilizing the open state of the K_v_7 channels. • Gabapentin is also a potent activator of K_v_7.2/7.3 heteromeric channels as well as K_v_7.3 and 7.5 homomeric channels. It does not act on homomeric K_v_7.2 and 7.4 channels • Zinc pyrithione binding occurs through a leucine residue in S5 and an alanine residue in the linker region between S5 and the pore. It has a high specificity of blocking K_v_7.2 homomeric channels (Xiong et al., 2008). • Flupirtine binds to all K_v_7 channels except K_v_7.2. • SCR2682 (IC_50_ of approximately 9.8 nM) is a K_v_7 channel activator, with a higher potency than retigabine. It causes a hyperpolarization shift of approximately 37 mV in the channel activation and can inhibit epileptic attacks in mice in a dose-dependent manner.	Main et al., 2000; Tatulian et al., 2001; Tatulian and Brown, 2003; Wickenden et al., 2009; Corbin-Leftwich et al., 2016; Xiong et al., 2008; Manville and Abbott, 2018; Barrese et al., 2018; Zhang et al., 2019

**TABLE 3:** The table summarizes various neurological anomalies dependent on the altered activity of the mAHP channels and their chemical modulators, which can alleviate the symptoms along with corresponding references.

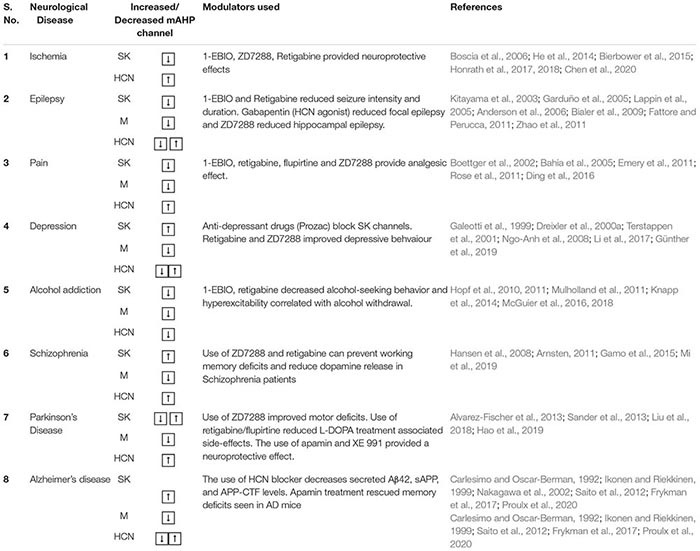

### Epilepsy

Epilepsy is a common neurological disorder caused by bursts of increased neuronal activity in different brain regions, which is characterized by jerking body movements. Because mAHP channels play a crucial role in controlling neuronal excitability, mutations in each of these channels can cause or aggravate epilepsy. Numerous studies have reported that a decrease in the activity of SK and M channels and an increase in the activity of HCN channels reduce neuronal mAHPs, which result in seizure phenotypes. Thus, mAHP channel modulators are potential therapeutic targets for epilepsy treatment.

In CA3 hippocampal cells, SK channels control epileptic bursts and spontaneous interictal discharge activity (Alger and Nicoll, 1980; Chamberlin and Dingledine, 1989; Fernández De Sevilla et al., 2006; Bakker et al., 2007). Ca^2+^ entry facilitated by L-type Ca^2+^ channels lead to the activation of these SK channels, which control burst duration, frequency, and intensity (Huang et al., 2018). In pilocarpine-treated chronic epilepsy rat models, a reduction in SK channel levels and currents has been observed (Schulz et al., 2012). Epileptic activity may cause different SK channel isoforms to undergo transient downregulation, further propagating acute seizures. In medial temporal lobe epilepsy (TLE) models, SK1 and SK2 channels and SK3 channel are downregulated acutely and chronically, respectively (Oliveira et al., 2010). Loss-of-function mutations in K_v_7.2 and 7.3 cause benign familial neonatal seizures, and G271V mutations in the pore region of K_v_7.2 cause familial epilepsy (Biervert et al., 1998; Wang et al., 2015). Increasing PIP2 levels by increasing PIP5K can rescue reduced K_v_7.2 function, thus suggesting that decreased “sensitivity” to PIP2 is responsible for impaired K_v_7.2 currents (Soldovieri et al., 2016). In specific benign familial neonatal seizures mutations, loss of preferential targeting of K_v_7.2/K_v_7.3 channels on AIS (due to mutation in the ankyrin G binding motif at the C terminal) and axons can cause losses in their function and epilepsy (Chung et al., 2006). Blocking of M channels increases ADP levels, which produces burst firing in hippocampal neurons, which start or propagate seizure waves and cause neurodegeneration (Vervaeke et al., 2006; Greene et al., 2018).

In contrast to SK and M channels, where reduced activity leads to epilepsy, an increase as well as a decrease in the activity of HCN channels are known to cause seizures. Increased HCN activity can reduce the mAHP of the neurons and produce hyperexcitability. Furthermore, reduced HCN levels increase cellular input resistance and dendritic excitability, which also causes hyperexcitability. This causes faster dendritic EPSC integration, which elicits spiking with fewer EPSCs. Increased dendritic excitability also increases EPSCs in the network, which increases network excitability (Biel et al., 2009). Genetic analysis of epilepsy patients revealed that the loss-of-function mutations in the HCN1 gene is responsible for various epilepsies, such as febrile seizures, genetic generalized epilepsies, and neonatal epileptic encephalopathy. Mutations in HCN2 and HCN3 can also contribute to epilepsy in some cases (Marini et al., 2018; DiFrancesco et al., 2019). In CA1 hippocampal neurons, 1–2 days after a single epileptic event, before the onset of spontaneous seizures, HCN1 channels exhibit an increase in surface expression. However, after 28 days, when spontaneous seizures have begun, the expression levels of HCN channel levels decrease. These bidirectional changes in HCN levels are responsible for the development of long-term epilepsy symptoms (Shin et al., 2008). Febrile seizures caused by fever are the most common type of seizures in children. A gain-of-function mutation in HCN2 is responsible for these seizures in patients (Dibbens et al., 2010). A hyperthermic event also produces an increase in incoming inhibitory post synaptic potentials (IPSPs), to reduce increased neural excitability during an epileptic activity. However, contrary to expectations, a burst of IPSPs, which is hyperpolarizing in nature, activates HCN channels, thereby causing hyperexcitability and increased seizure attacks (Chen et al., 2001). Contrary to the aforementioned studies, a single seizure episode can reduce HCN1 and HCN2 channel currents after 24 h for up to 1 week in EC layer III and hippocampal neurons (Shah et al., 2004; Jung et al., 2007; Powell et al., 2008). Additionally, loss of function in HCN2 and loss of TRIP8b protein (an auxiliary protein to HCN channels) can lead to the emergence of spontaneous absence seizures (Ludwig et al., 2003; Heuermann et al., 2016). TRIP8b deletion mice have reduced I_h_ currents which can cause loss of HCN-mediated inhibition of T-type calcium currents in the thalamocortical relay and cortical neurons. This leads to increased spontaneous oscillatory activity in the thalamocortical neuronal networks and subsequent spontaneous absence seizures in mice (Ludwig et al., 2003; Ramírez et al., 2018; Hammelmann et al., 2019; Zobeiri et al., 2019). In the TLE model of epilepsy, reduced phosphorylation of Ser^237^ in TRIP8b proteins has also been found to be a contributing factor to epilepsy (Foote et al., 2019). Loss of KCNE2 can also lead to reduced levels and slowed activation kinetics of HCN channels (Ying et al., 2012).

Because a decrease in SK and M channel activity and an increase or a decrease in HCN channel activity causes epilepsy symptoms, their modulators are useful therapeutic targets. The use of SK and M activators, 1-EBIO, and retigabine reduce epileptic activity. Additionally, they delay seizure initiation; increase the threshold; reduce seizure intensity in the kindling model of epilepsy, audiogenic seizures, and focal epilepsy; and reduce seizure intensity in genetically epilepsy-prone mice. However, they also cause side effects such as reduced locomotor activity, dizziness, fatigue, loss in motor coordination, and impairment in exploratory behavior (Xing and Hu, 1999; Garduño et al., 2005; Lappin et al., 2005; Anderson et al., 2006; Bialer et al., 2009; Fattore and Perucca, 2011; Weisenberg and Wong, 2011). Augmenting K_v_7 activity after an epileptic event also prevents neurodegeneration (Greene et al., 2018). In genetically epilepsy-prone rat models, acoustic seizures were reported to reduce the activity of SK1 and SK3 channels in the inferior colliculus, and the use of 1-EBIO was reported to reduce seizure incidences in these models (Khandai et al., 2020; Table 2). D-23129, a derivative of flupirtine, is also effective against amygdala kindling seizure models (Tober et al., 1996). HCN1 channels limit the spread of seizures from the forebrain to the hindbrain (Huang et al., 2009; Santoro et al., 2010). Because an increase/decrease in the levels or activity of HCN channels can contribute to epilepsy, both blockers and activators of HCN channels are possible therapeutic agents. Gabapentin, an HCN channel agonist, is reported to be effective against focal epilepsy, while the HCN channel blocker ZD 7288 reduces hippocampal epileptic activity in rabbits (Kitayama et al., 2003; Table 2). In the VB nucleus, loss of HCN2, but not HCN4, alters underlying thalamic oscillations, thereby causing absence seizures (Ludwig et al., 2003; Hammelmann et al., 2019; Zobeiri et al., 2019). By contrast, David et al. (2018) reported a decrease in I_h_ levels in the neocortical neurons in absence seizure models and an increase in I_h_ currents in the thalamocortical neurons. Microdialysis of ZD7288 in the VB nucleus or knockdown of HCN channels in the VB nucleus abolishes absence seizures (David et al., 2018). Bidirectional changes in HCN levels in different brain regions negates the possibility of treating epilepsy symptoms by using a general HCN blocker. Additionally, SK channels contribute to alterations in thalamocortical oscillations. In a bicuculline methiodide epilepsy model, the SK channel antagonist apamin was reported to produce epileptic thalamocortical oscillations, which are the underlying causes of absence seizures. Thus, both HCN and SK channels are involved in the etiology of absence seizures and their dual modulation is likely an effective treatment strategy (Ludwig et al., 2003; Kleiman-weiner et al., 2009; Hammelmann et al., 2019).

As explained previously, mAHP channels are critical molecules contributing to epilepsy. The levels of SK and HCN channels increase and decrease in an isoform-specific and a time-dependent manner, which result in epilepsy initiation and progression. Loss-of-function mutations in Kv7 channels can also lead to epilepsy. Additional work to elucidate region-dependent isoform-specific alterations in the levels of the mAHP channels can be effectively used in clinical practice (Table 3).

### Pain

Dorsal root ganglion, peripheral neurons, sensory neurons, and nociceptor neurons express all the three mAHP channels (Akins and McCleskey, 1993; Villière and McLachlan, 1996; Amir and Devor, 1997; Boettger et al., 2002; Passmore et al., 2003; Linley et al., 2008; Hou et al., 2015). These channels regulate the firing activity of the aforementioned neurons, thereby modulating pain responses. Pain or nerve damage reduces mAHP channel activity prompting hyperactivity in these neurons; thus, mAHP channel activators can be used as pain-relieving agents.

Injury to a peripheral neuron or its nearest DRG leads to ectopic spontaneous discharges on account of diminished extracellular potassium or calcium (Xing and Hu, 1999). Xing and Hu (1999) showed that increasing extracellular calcium increases calcium influx, activates SK currents, and reduces firing in DRG neurons. Thus, activating SK channels eases pain perception. Reduction in SK1 and K_v_7.2, 7.3, and 7.4 currents and increased HCN activity in small and large nuclei DRG neurons after injury contribute to increased neuronal activity and pain sensation (Boettger et al., 2002; Emery et al., 2011; Rose et al., 2011; Yu et al., 2018). Elevated HCN currents in rat thalamus and spinal cord present chronic neuropathic pain. Similarly, HCN2 knockout mice showed a reduction in pain response to thermal or mechanical pain stimuli (Emery et al., 2011). In the spared nerve injury pain model, the medial prefrontal cortex (mPFC) neurons showed reduced cAMP levels and loss in PKA activity leading to a hyperpolarization shift in HCN activity (Matos et al., 2015). Although not examined in this study, loss in PKA-dependent regulation will also reduce M channel activity and SK channel surface expression (Lin et al., 2008; Abiraman et al., 2016; Mani et al., 2016). The combined loss of activity of mAHP channels can give rise to increased IR and hyperexcitability in these neurons, thereby increasing pain sensation (Matos et al., 2015). Neuropeptide S is a naturally occurring anxiolytic that inhibits HCN channels on medial amygdala cells. Reducing HCN activity by using neuropeptide S enhances glutamatergic inputs on GABAergic interneurons, which in turn suppresses pyramidal cells and relieve pain (Zhang et al., 2016). Neuropeptide S also possesses weak capacity to bind to SK channels (Liegeois et al., 2005). Mimicking neuropeptide S action and inhibiting HCN and SK channels by targeting amygdalar nuclei is a possible pain-relieving mechanism.

Additionally, blocking HCN channels by injection of ZD7288 in the thalamus has shown to attenuate chronic pain and sensitivity to pain in a dose-dependent manner (Ding et al., 2016; Zhang et al., 2016) (Table 2). Propofol (2,6-di-isopropyl phenol) and its derivatives, 2,6- and 2,4-di-tertbutylphenol are potent, selective HCN1 channel blockers and can reduce mechanical and thermal hyperalgesia (Tibbs et al., 2013). Furthermore, chronic pain also contributes to anxiety. Koga et al. (2015) found that an increase in the surface expression of HCN channels on the anterior cingulate cortex neurons increases both pain and anxiety. The use of ZD7288 reduces pain perception. HCN blockers, such as ZD7288, loperamide, and clonidine, also inhibit spontaneous neuronal discharge and ectopic spontaneous firing activity from DRG neurons in the event of a nerve injury, thus producing analgesic effects (Yagi and Sumino, 1998; Vasilyev et al., 2007; Bernal and Roza, 2018) (Table 2). Ivabradine is an FDA-approved drug that blocks all HCN channel isoforms. The drug can provide prolonged relief from trigeminal neuropathic pain by acting on the peripheral nervous system (Young et al., 2014; Ding et al., 2018). However, a clinical trial conducted by Lee et al. (2019) showed ivabradine reduces the heart rate without significant analgesic effects in human capsaicin-induced pain models.

In the spinal nerve ligation pain model, reducing SK channel activity in the central amygdala output nuclei was reported to increase pain sensitivity. SK channel activation by riluzole or 1-EBIO can reduce sensory inputs to the spinal cord and produce analgesic effects (Bahia et al., 2005; Thompson et al., 2018). M channel blockers, such as retigabine and flupirtine, can also relieve pain symptoms, as seen in chronic constriction injury, spinal cord injury, and nerve damage pain models (Blackburn-Munro and Jensen, 2003; Devulder, 2010; Rose et al., 2011; Wu et al., 2017; Yu et al., 2018; Table 2). Non-steroidal anti-inflammatory drugs, such as meclofenamate, diclofenac, NH6, and NH29 and acetaminophen (paracetamol), relieve migraine and neuropathic pains by activating M channels. These drugs produce a hyperpolarization shift in M channel activation and reduce their inactivation (Peretz et al., 2005, 2007, 2010; Ray et al., 2019). Artificially synthesized M channel activators, such as pyrazolopyrimidines, benzimidazole, and pyrazolo[1,5-a] pyrimidine-7(4H)-one compounds, have enhanced potency and diminished side effects (Wickenden et al., 2009; Zhang et al., 2011; Zhang F. et al., 2013; Osuma et al., 2019). QO-58 and QO-lysine are the pyrazolo[1,5-a] pyrimidine-7(4H)-one compounds that can alleviate pain symptoms in the chronic constriction injury pain model by activating all K_v_7 isoforms except for K_v_7.3 (Zhang M. et al., 2013; Teng et al., 2016). Paclitaxel treatment in patients with cancer produces hyperexcitability in nociceptive neurons, which contributes to chronic pain. Early treatment with retigabine can avert chronic pain (Li et al., 2018). Future investigations are focusing on finding K_v_7 channel activators, targeting the peripheral nervous system and averting the side effects caused by their action on the central nervous system (Hayashi et al., 2014). Reduced activity of SK, M and increased activity of HCN channels causes hyperexcitability in the sensory or nociceptive neurons, which increases pain sensation. Specific targeting and increasing the activity of the mAHP channels in the pain perception neurons can produce analgesic effects (Table 3).

### Depression

An imbalance in the dopamine (DA) levels in the ventral tegmental area (VTA)–Nucleus accumbens (NAc) circuit in the underlying cause of depression and anxiety behavior (Polter and Kauer, 2014). DA neurons exhibit a wide range of firing patterns from tonic spiking pace-making activity to a bursting response. These diverse firing activities or patterns control dopamine release in the brain (Paladini et al., 2003). Stress and depression can increase or reduce firing in the VTA DA neurons, depending on the severity of the stress. VTA DA neurons exhibit an increase in firing under severe stress conditions and a decrease in firing when subjected to weak stress (Valenti et al., 2012).

Alterations in SK, HCN, and M channel activity cause altered neuronal firing activity, leading to significant stress or depression symptoms. An increase in SK activity, a reduction in M channel activity, and an increase or decrease in HCN activity conductance can produce depression symptoms in mice. Hippocampal-specific HCN4 knockdown causes increased anxiety, whereas an increase in perisomatic HCN1 channels in CA1 hippocampal neurons contribute to chronic stress. These findings demonstrate the isoform-specific but opposing effects on a given phenotype within the same brain region (Kim et al., 2017; Kim and Johnston, 2018; Günther et al., 2019). Chronic stress causes an increase in L-type Ca^2+^ channels and increased intracellular calcium through CICR (Kim et al., 2017; Kim and Johnston, 2018). Increased calcium levels augment both SK and HCN channel activity and reduce M channel activity. Enhanced SK and HCN channel activity and reduced M channel activity contribute to increased tonic firing activity in VTA DA neurons, which is associated with depressive behavior. SK channel antagonists can transform tonic firing activity to phasic firing activity in DA neurons, which can rescue antidepressive behavior in mice (Van Der Staay et al., 1999; Oster et al., 2015). In the social defeat stress model, VTA neurons exhibit increased excitability due to a reduction in the expression levels of M channel and an increase in HCN activity. The overexpression of K_v_7.2, retigabine injection in VTA, chronic treatment with fluoxetine (HCN and SK channel antagonist), or local infusion of ZD7288 have been reported to improve social behavior, reduce anxiety, increase sucrose preference, and produce antidepressive behavior in mice (Cao et al., 2010; Friedman et al., 2016; Li et al., 2017) (Table 2).

Multiple antidepressant drugs, including tricyclic antidepressants, such as desipramine, imipramine, and nortriptyline, as well as phenothiazines, such as fluphenazine, promethazine, chlorpromazine and trifluoperazine, and apamin, can block SK2 and SK3 channels, thereby reducing immobility time in the forced swim test, which is a model for depression (Galeotti et al., 1999; Dreixler et al., 2000b; Terstappen et al., 2001) (Table 2). Fluoxetine (prozac) is a common serotonin reuptake inhibitor that is used to treat depression, anxiety, and obsessive–compulsive disorders. It can block all three isoforms of SK channels to varying degrees (Terstappen et al., 2003). In addition to the VTA DA circuit, increased activity in the amygdala in response to fear stimuli also contributes to anxiety in animals. The use of the M channel agonist, BMS-204352, and retigabine exhibits an anxiolytic effect on these mice (Rauch et al., 2003; Korsgaard et al., 2005) (Table 2).

Additionally, alterations in HCN channels, but not in SK and M channels, can increase resilience to depression and facilitate coping with stress (Fisher et al., 2018). Contrary to expectations, mice that are resilient to depression were reported to exhibit higher HCN currents than those with depression, but the resilient mice did not exhibit increased firing in VTA neurons. This increased HCN channel activity probably activates some other K^+^ conductance, which restores the firing activity in resilient mice to a level comparable to that of the control mice. Mice with TRIP8b deletion, which causes reduced neuronal expression of HCN channels, and those with HCN1 knockdown exhibit resilience to depressive behavior (Lewis et al., 2011; Kim et al., 2012; Han et al., 2017). Thus, mAHP channels strongly affect the net dopamine level in the brain and control the activity of amygdala neurons, which control emotions in an animal. Hence, mAHP channel modulators potentially exhibit both antidepressive action and can cause an increase in resilience to depression (Table 3).

### Alcohol Addiction

Altered neurotransmission, abnormal molecular pathways, and aberrant ion channel activity can give rise to alcohol addiction and withdrawal relapse. Alcohol addiction involves alcohol-seeking behavior, anxiety during alcohol withdrawal, alcohol-seeking behavior during withdrawal, and alcohol extinction memory. The aforementioned behavioral decisions are controlled by different brain regions. Altered neuronal excitability due to the mAHP channels affects the functioning of these regions.

Ethanol consumption increases DA neuronal activity in the substantia nigra (SN) and VTA because of the alteration of HCN, M, and SK channel activity (Hopf et al., 2007; Cannady et al., 2017; Rinker et al., 2017). SK channels coupled to NMDA channels, protect against NMDA-dependent excitotoxicity. Chronic ethanol consumption produces NMDA–SK functional uncoupling causing hyperexcitability in CA1 cells (Mulholland, 2012), reduced HCN currents in VTA neurons, and reduced K_v_7.2/7.3 surface expression in nucleus accumbens (NAc) neurons (Hopf et al., 2007; McGuier et al., 2016). VTA DA neurons undergo a transition in firing activity from their regular pace-making activity to burst firing during the withdrawal phase, which is correlated with relapse. A reduction in SK2 levels, which causes NMDAR hyperactivity, was reported to be responsible for this transformation in firing patterns (Hopf et al., 2007; Mulholland, 2012). NAc is correlated with drug-induced addiction and reward systems (Janak et al., 1999). After alcohol abstinence, the NAc core neurons become hyperexcitable, which increases alcohol-seeking behavior. This hyperexcitability results from reduced activity of SK3 channels (but not SK2 channels) and reduced K_v_7.2/7.3 surface expression levels in NAc neurons (Hopf et al., 2011; McGuier et al., 2016). Systemic retigabine, a microinfusion of retigabine in NAc and VTA DA neurons, retigabine administration in rats, and the use of chlorzoxone, an SK agonist, have been reported to reduce firing in NAc neurons as well as ethanol consumption (Hopf et al., 2011; Knapp et al., 2014; McGuier et al., 2016, 2018). The use of an SK activator (1-EBIO) is also reported to reduce motivation for alcohol (Hopf et al., 2010). Furthermore, 1-EBIO reduced network hyperexcitability and neurotoxicity associated with ethanol withdrawal in mice (Mulholland, 2012; Table 2).

Increased lateral habenula activity due to a decrease in K_v_7.2/7.3 channel activity causes alcohol withdrawal–driven anxiety. Inhibition of habenula by the M channel agonist retigabine rescued anxiety phenotypes and alcohol preference in tested animals (Kang et al., 2017). The inhibition of serotonergic pathways can also treat alcohol withdrawal–associated anxiety. Inhibiting serotonergic pathway increases K_v_7.2/7.3 channel expression, which reduces firing in lateral habenula neurons and helps treat alcohol addiction (Fu et al., 2020). In addition to the use of SK activators, which can restore regular firing activity in VTA, NAc, and hippocampal neurons and assist in alcohol abstinence, SK blocker treatment in the infralimbic prefrontal cortex causes alcohol extinction memory (Cannady et al., 2017). mGluR5 and SK2 are coupled functionally and act collectively to regulate neural excitability (Sourdet et al., 2003). Increasing mGluR activity aids in alcohol and cocaine-seeking extinction learning (Gass and Olive, 2009; Cleva et al., 2011; Cannady et al., 2017). Increased mGluR activity downregulates SK2 channels in the infralimbic prefrontal cortex, contributing to extinction learning. Microinfusion of the SK blocker apamin in the infralimbic prefrontal cortex aids in the process of extinction learning (Cannady et al., 2017). Thus, the use of an SK activator can control neuronal hyperexcitability due to alcohol withdrawal, and SK inhibitors can facilitate extinction learning. The dual effect of SK channel activators and inhibitors highlight the heterogeneity of SK channel operating in diverse brain regions, which further emphasizes the need for directed channel inhibition and activation in various brain regions to alleviate diverse disease symptoms (Table 2). In summary, excessive alcohol consumption leads to addiction, and withdrawal causes anxiety owing to changes in the firing pattern of VTA, SN, NAc, and habenula neurons, which in turn affect the dopamine levels in the brain. The use of SK and M activators can restore regular firing activity and help overcome addiction (Table 3).

### Schizophrenia

Schizophrenia is a degenerative mental disorder characterized by hallucinations, anxiety, and eccentric behavior. Like depression, alterations in brain dopamine levels contribute to the pathophysiology of schizophrenia. Genetic mutations and environmental components can trigger and aggravate schizophrenia symptoms.

Midbrain DA neurons exhibit high levels of expression of SK3 and K_v_7.2/7.4 homomeric channels, which control their firing patterns and hence dopamine release (Rimini et al., 2000; Stocker and Pedarzani, 2000; Hansen et al., 2008). Alterations in SK and M channel activity can cause an imbalance in dopamine levels in the brain, thereby contributing to schizophrenia symptoms such as hallucinations (Hansen et al., 2008). An increase in CAG repeats in the SK3 gene with a higher polymorphism in the repeat length of CAG increases susceptibility to schizophrenia (Bates and Davies, 1997; Chandy et al., 1998; Wittekindt et al., 1998; Cardno et al., 1999). Increased glutamine (because of increased CAG numbers) contributes to a gain-in-function mutation in SK channels by either enhancing the channel’s calcium sensitivity or modifying its kinetic properties. In maternal immune activation and juvenile social isolation mouse models of schizophrenia, layer 5 PFC neurons had higher expression levels of SK3 channel (with no change in SK1 and SK2 expression levels), which cause depolarized cellular RMP, higher levels mAHP expression, and schizophrenia-related behavioral deficits (Mi et al., 2019). By contrast, some other studies have either reported a lack of association between SK3 gene CAG repeat polymorphism and schizophrenia or have observed loss-of-function variants in the SK3 gene. A frameshift mutation in exon 1 of the SK3 gene produced a truncated form of the protein that did not exhibit transmembrane segments, was accumulated in the nucleus, and was not express on the cell surface (Bowen et al., 2001; Miller et al., 2001; Laurent et al., 2003; Ritsner et al., 2003; Tomita et al., 2003). Linkage studies have also demonstrated an association between SK3 gene polymorphism and mutations in phosphatidylinositol-4-phosphate 5-kinase II alpha in patients with schizophrenia (Wittekindt et al., 1998; Brzustowicz et al., 2000; Bakker et al., 2007). Phosphatidylinositol-4-phosphate 5-kinase II alpha mediates PIP2 synthesis. Loss of function of phosphatidylinositol-4-phosphate 5-kinase II alpha, as observed in patients with schizophrenia, reduces PIP2 levels, which reduces M channel activity (Bakker et al., 2007; Fedorenko et al., 2008). Disrupted M channel activity in neurons in the SN and VTA DA alters brain dopamine levels. K_v_7.4 channel enhancers, such as retigabine, can increase M channel activity in DA neurons and can alleviate schizophrenia symptoms (Hansen et al., 2008; Peng et al., 2017).

Neuronal NMDAR hypofunction is one of the most promising theories explaining the pathophysiology of schizophrenia. In support of the NMDAR hypofunction theory, the use of phencyclidine, an NMDAR blocker, was reported to produce psychotic schizophrenia-like symptoms in experimental animals (Lodge and Anis, 1982; Davies et al., 1988; Javitt, 1991). Increased SK activity can cause NMDAR hypofunction (Bates and Davies, 1997; Chandy et al., 1998; Gargus et al., 1998). Reduced SK2 channel activity in DA neurons produces bursting firing activity, which increases dopamine levels in the brain, contributing to schizophrenia symptoms. Hence, blocking dopamine receptors is an approved remedy for patients with schizophrenia (Olney and Farber, 1995; Farde, 1998; Lam et al., 2013). SK2 activators also reduces dopamine release and might alleviate some symptoms. However, the use of SK2 activators will increase NMDAR hypofunction and aggravate the symptoms. Thus, additional meticulous investigation to assess the role of SK channels in schizophrenia is crucial for understanding its therapeutic potential. However, selective blocking or activating specific SK isoforms in distinct brain regions, while sparing others, is a potential therapeutic strategy.

Altered neuronal activity in the PFC, which regulates the working memory, also contributes to schizophrenia symptoms. Disrupted in schizophrenia 1 (DISC1) protein is a cAMP effector protein; low levels of DISC1 are correlated with schizophrenia (El-Hassar et al., 2014). In PFC pyramidal neurons, diminished levels of DISC1 cause an increase in mGluR-dependent increase in intracellular calcium influx, which augments SK channel activity (El-Hassar et al., 2014). In PFC neuronal spines, DISC1, along with other cAMP effector proteins, such as PDE4 and D1Rs, are also co-localized with HCN channels. DISC1 regulates cAMP levels; hence, its loss of function, as observed in schizophrenia, causes increased cAMP levels, which in turn increase HCN channel activity and lead to spine loss (Arnsten, 2011; Paspalas et al., 2013). Stress is another factor, which can act as a stimulant or exaggerator of schizophrenia symptoms. Increased stress can elevate cAMP and PKA levels through D1R activation, which further increases HCN, and probably M channel activity; both cooperatively reduce firing activity and the number of spines in PFC neurons (Arnsten, 2011). A specific HCN blocker, ZD7288, can rescue this reduced firing response in PFC and prevent working memory deficits (Gamo et al., 2015; Table 2). Positron emission tomography imaging studies in patients with schizophrenia have shown increased D1R binding in PFC neurons, and the use of effective antipsychotic drugs has been shown to reduce D1R expression (Abi-Dargham et al., 2002; Hirvonen et al., 2006). Thereby, region-specific gain-of-function mutations of SK channels and reduced PIP2 levels that diminish M channel function cause abnormal dopamine levels in the brain circuits. Additionally, increased HCN channel activity contributes to hyperactivity in DA neurons. Thus, use of mAHP channel modulators can restore dopamine levels and firing activity of specific neurons, thereby alleviating schizophrenia symptoms (Table 3).

### Parkinson Disease

Parkinson disease (PD) is a neurodegenerative disease characterized by loss of DA neurons in the substantia nigra (SN) and basal ganglia because of α-synuclein aggregates called Lewy bodies. This loss of function of DA neurons reduces brain dopamine levels and causes ataxia, tremors, and motor disability, the common symptoms of PD. PD is associated with the dysfunction of multiple ion channels (Pedarzani and Stocker, 2008; Wang et al., 2008). SK, HCN, and M channels present in DA neurons and striatal cholinergic interneurons control the firing activity of DA neurons. The single spiking tonic firing mode of these neurons is reported to control baseline dopamine levels, while their transition to burst firing activity increases their postsynaptic dopamine release (Pedarzani and Stocker, 2008; Carbone et al., 2017). SK, HCN, and M channel blockers can alleviate PD symptoms and provide neuroprotective effects, while M activators reduce the side effects associated with levodopa (L-DOPA) treatment.

In a rat model of PD, SK3, and SK2 exhibited opposite trends of change. In the SN, downregulation of SK3 channels is observed with disease progression, while in the subthalamic nucleus (STN), a delayed increase in SK2 levels (21 days after PD induction) was reported. Furthermore, differential effects of blocking these SK channels were reported in the SN and STN. Blocking SK channels by using apamin in the substantia nigra pars compacta (SNc) alleviated PD symptoms, while the symptoms were aggravated when the channels were blocked in the STN (Mourre et al., 2017). Initial PD symptoms were reported to correlate with changes in beta frequency oscillations in globus pallidus (GPe) neurons. In healthy brains, the activities of different GPe neurons are unsynchronized. However, upon dopamine depletion in PD GPe neurons are unable to maintain their pacemaking activity leading to increased intraneuronal oscillations within basal ganglia with a sharp peak in the beta frequency range. Alteration in HCN and SK channels have been reported to be responsible for GPe neuron synchronization and changes in network properties (Chan et al., 2011; Schwab et al., 2013). Microinjection of the HCN blocker ZD7288 *in vivo* in the GPe increases the firing activity of some neurons and reduces firing in some others. Overall, ZD7288 improves motor deficits observed in 1-methyl-4-phenyl-1,2,3,6-tetrahydropyridine) PD mouse models (Hao et al., 2019). In PD rat models, many DA neurons were reported to undergo degeneration, and the surviving DA neurons exhibited a reduction in HCN channels, which altered their firing pattern (Guatteo et al., 2017). Downregulation of HCN channels by multiple mechanisms contribute to various PD symptoms.

SUMOlytation of α-synuclein helps in correct protein folding and produces soluble protein. By contrast, deSUMOlytation of α-synuclein causes their aggregation leading to PD. Impaired SUMOlytation can also lead to a reduction in surface levels of HCN channels in PD (Parker et al., 2017). Progression of PD also correlates with mitochondrial dysfunction, which in turn increases oxidative stress on the cell and reduces ATP levels in the cell. This produces low cAMP levels and reduced HCN functions (Schapira and Gegg, 2011; Carbone et al., 2017). Reduction in HCN channel transcription produces a spike-timing impairment in Ach-releasing striatal interneurons (Chl), which reduces the net Ach release. Although both Ach and DA levels are low in PD brains, surviving Chl neurons increase their Ach release, which leads to an increase in the Ach/DA ratio and contributes to PD symptoms (McKinley et al., 2019). K_v_7.2, 7.3, and 7.5 expressed on striatal neurons and Chls can regulate their activity and excitability.

The use of artificial L-DOPA is a long-term treatment strategy for PD (Dunnett and Björklund, 1999). It compensates for the lack of DA and mitigates motor deficit. However, its chronic use leads to impairment of voluntary movement (dyskinesia) and increased severity of involuntary movement (Sander et al., 2012). The administration of K_v_7 channel openers retigabine and flupirtine along with L-DOPA has been reported to reduce the symptoms of dyskinesia without influencing the effect of L-DOPA (Sander et al., 2012; Pérez-Ramírez et al., 2015). A specific activator of K_v_7.2/7.3, ICA27243, exhibits a substantial antidyskinetic effect, without the side effects of sedation and ataxia, which are observed with the use of retigabine. The beneficial effects were because of specific targeting of striatal neurons only, which spared other channel isoforms (Sander et al., 2013).

SK and M channel blockers have shown neuroprotective activity in different PD models (Salthun-Lassalle et al., 2004; Doo et al., 2010; Alvarez-Fischer et al., 2013; Paz et al., 2018; Table 2). Blocking M channels by using XE 991 prevents the loss of DA neurons, increases the activity of surviving striatal DA neurons, restores dopamine levels, and improves motor coordination (Liu et al., 2018). Thus, where M channel activators can facilitate circumventing the side-effects of L-DOPA treatment, M channel blockers serve a neuroprotective role. Both M and SK blockers cause an increase in intracellular Ca^2+^ levels, which increases DA neuron excitability. In the 1-methyl-4-phenyl-1,2,3,6-tetrahydropyridine-induced PD model, bee venom (apamin) acupuncture in limbs or intraperitoneal administration of apamin prevented the loss of DA neurons in the SN and striatum, thus evidencing its neuroprotective action. Moreover, in an *in vitro* mesencephalic neuronal culture, the use of apamin prevented neuronal degeneration (Salthun-Lassalle et al., 2004). The use of bee venom to block SK activity has also been reported to cause an increase in basal dopamine levels in the striatum, which reduces motor deficits, prevents catalepsy symptoms, and causes a general increase in motor activity in PD mouse models (Alvarez and Sabatini, 2007; Chen et al., 2014; Maurice et al., 2015). Bee venom exhibits neuroprotective effects because it downregulates Jun N-kinase activity, which is responsible for the degenerative effects observed in 1-methyl-4-phenyl-1,2,3,6-tetrahydropyridine-induced PD models (Doo et al., 2010; Alvarez-Fischer et al., 2013). Other studies have also shown that Jun N-kinase activity causes neurodegenerative effects in PD animal models and reducing its activity can be a therapeutic strategy. Patients with PD and animal models exhibit an increase in Jun N-kinase activity (Hunot et al., 2004).

Contrary to the aforementioned results, where SK blockers were reported to provide a neuroprotective effect and increase in basal DA levels, an organotypic PD culture of the SN was reported to exhibit rescue effects in the presence of 1-EBIO, an SK channel activator. The SN cells exhibited a change in firing behavior from their regular pace-making to an irregular bursting activity. Furthermore, the cells exhibited an increase in resting membrane potential and neurotoxic death. 1-EBIO reduced burst firing and prevented the 6-OHDA mediated death of these neurons (Wang et al., 2014). Thus, contradictory information is available to answer the question of whether a blocker or an enhancer of SK channels has therapeutic potential. Possibly, SK channels influence both excitability and spiking patterns; consequently, they have a complex effect on the disease pathophysiology. Therefore, a detailed study is necessary to understand the contribution of SK channels to PD and how their modulation might alleviate PD symptoms. A clinical trial that used bee venom, patients with PD did not show improvement. However, the authors believe that the use of higher levels of the toxin at an increased frequency might result in an improvement in the symptoms (Hartmann et al., 2016).

As with other neurological disorders, region-dependent alterations in SK channel expression levels are observed, such that blocking SK channels in the SNc but not in STN alleviates PD symptoms. An M channel opener can circumvent side effects of L-DOPA treatment, and an HCN channel opener can be used to restore dopamine/Ach ratios. A drug cocktail modulating all mAHP channels can potentially be used to treat multiple PD symptoms (Table 3).

### Alzheimer’s Disease

Aggregation of insoluble Aβ fibrils in the brain is the principal underlying cause of Alzheimer’s Disease (AD). Amyloid precursor protein (APP) cleaved by β secretase yields a carboxy-terminal fragment (APP-CTFβ) and an amino-terminal soluble APP fragment (sAPPβ). Furthermore, abnormal APP-CTFβ cleavage by γ secretase (presenilin) yields Aβ42 and APP intracellular domain (AICD). Aβ42 undergoes fibril aggregation, causing progressive neurodegeneration, while AICD modulates intracellular calcium signaling assisting in aggregate formation (Leissring et al., 2002; Vassar et al., 2014; Frykman et al., 2017). Inhibition of β and γ secretase is the most promising contender for AD treatment. However, β and γ secretase inhibition can also influence other cellular pathways, producing severe side effects. For example, β secretase augments M channel activity. Reducing β secretase activity will also inhibit M currents, which might aggravate seizure phenotypes in patients with AD (Vossel et al., 2013; Vassar et al., 2014; Hessler et al., 2015).

According to the calcium hypothesis proposed by Khachaturian (1989), intracellular calcium dysregulation contributes to the phenotypes associated with AD. Studies have demonstrated that changes in calcium dynamics assist in Aβ fibril formation, and Aβ fibril aggregation further increases calcium influx, which increase neurological deterioration. A study by Yoo et al. (2000) showed that reduced calcium entry is an early event in the Presenilin AD model and facilitates Aβ fibril formation. Furthermore, fibril aggregation increases G protein-coupled receptor-mediated CICR, which increases intracellular calcium levels and contributes to neurotoxicity (Leissring et al., 2002; Stutzmann, 2005). Human studies have shown that an increase in intracellular calcium influx induced by Aβ fibril aggregation increases the vulnerability of cortical cells to calcium-mediated neurotoxicity (Mattson et al., 1992). Increased intracellular Ca^2+^ levels can cause increase SK channel activity, leading to memory deficit, which is observed in AD mouse models and patients with AD (Carlesimo and Oscar-Berman, 1992). A study provided evidence of an age-dependent increment in SK channel activity, which can lead to further aggravation of AD symptoms (Blank et al., 2003). Thus, apamin treatment has been reported to reduce deficits in spatial learning and working memory in AD mice (Ikonen and Riekkinen, 1999; Proulx et al., 2020). Increased calcium influx also reduces PIP2 binding to M channels and diminishes their kinetics. Additionally, reduced expression levels of KCNE5 (auxiliary protein to K_v_7 channels, which assist their activity) reduces the activity of K_v_7.3 and K_v_7.5 channels in cerebral arteries in the AD mouse model. Consequently, blood flow in the cerebral areas decreases, promoting fibril aggregation (de Jong and Jepps, 2018).

The effect of HCN channels on AD pathophysiology is not well understood. Both HCN1 upregulation and HCN2 blocking can be useful in treating AD pathophysiology. In AD mouse models and in the brains of patients with AD, HCN1 channel expression is reduced (Saito et al., 2012). In AD mouse models, aging causes HCN1 channels to get sequestered in the endoplasmic reticulum, thereby reducing net HCN channel expression on the distal dendrites of CA1 neurons and resulting in CA1 hyperexcitability and seizure phenotypes (Musial et al., 2018). X11 and X11L are the proteins that assist in Aβ trafficking. HCN channels can form a complex with X11, X11L, and APP. Mutant mice lacking X11 and X11L were reported to exhibit a higher incidence of seizure because of reduced HCN channel conductance. HCN1 knockout mice and mice with HCN1 blocking were reported to exhibit increased Aβ aggregation (Saito et al., 2012). Thus, reduced HCN channel expression contributes to AD symptoms. Consistent with the aforementioned results, treatment of Aβ aggregation in CA1 hippocampal cells caused increased HCN1 expression and reduced neuronal excitability (Eslamizade et al., 2015). However, contrary results were obtained in another study; blocking HCN2 channels in HEK cells reduced secretion of Aβ42, sAPP, and APP-CTF by reducing APP maturation or β action (Frykman et al., 2017). The differential effects may be isoform-specific effects or part of underlying signaling changes, the details of which warrant examination.

AD involves complex changes in intracellular calcium dynamics, which directly or indirectly affect all three mAHP channels. Increased SK currents, reduced M currents and increased or decreased HCN currents in neurons and cerebral arteries control fibril aggregation, seizure incidence and memory deficits. Thus, mAHP channel modulators can potentially alleviate some AD symptoms (Table 3).

## Conclusion

The therapeutic potential of mAHP channels is being explored recently for treating various neurological anomalies. Decades of research have provided us with extensive information about the heterogeneity of channel isoforms and their expression in various brain regions. The current review highlights the commonalities and specializations of the three mAHP channels. Although the three channels work together to reduce excessive neuronal firing, share similar subcellular and regional localization, are controlled by similar regulatory pathways, and exhibit similar activation and inactivation kinetics, they have region-specific isoforms that are responsible for specialized neuronal functions. The concept of isoforms is an essential point of consideration for future research because it stresses the point that an indiscriminate modulation of an ion channel might produce undesirable effects. For example, in schizophrenia, M channel activity decreases in DA neurons and increases in PFC neurons, causing reduced spiking response and spine loss, respectively. The use of an M channel activator to rescue firing activity in DA neurons aggravates the spine loss phenotype of PFC neurons. Similarly, an M channel blocker rescues the spine loss phenotype in PFC neurons, but it further reduces DA firing activity. Similarly, in the PD model, SK channels in SNc and STN neurons exhibit opposite trends. In SNc neurons, a reduction in the levels of SK3 is observed, while in STN neurons, a delayed increase in SK2 levels is observed. Thus, the use of a general activator or blocker may have limited clinical potential. In the worst case, the general activator or blocker may aggravate disease symptoms. Consequently, isoform- and region-specific modulation is needed to provide an effective treatment strategy. Multiple other examples cited in this review clearly illustrate isoform- and region-specific alteration in the activity of various mAHP channel, which demands the identification, synthesis, and use of isoform- and region-specific modulators. Research in the past few years appears to be focused on the synthesis of several isoform-specific modulators. Additionally, intervention timing might be another crucial factor in treating various neurological diseases. Studies have shown transient as well as long-term changes in mAHP channel levels, emphasizing the need for extensive information on age-dependent and region-dependent alterations to identify the correct intervention that can be used to treat disease symptoms. In addition to isoform-specific modulators, region-specific gene targeting to remedy gain- or loss-of-function mutations in mAHP channels can also be used to treat abnormal neurological functions. With the advancement of region-specific targeting techniques, we might witness a revolution in treatment methodology in the near future.

## Author Contributions

DD wrote the manuscript. Both authors structured and refined the manuscript.

## Conflict of Interest

The authors declare that the research was conducted in the absence of any commercial or financial relationships that could be construed as a potential conflict of interest.
